# All‐in‐One Compression and Encryption Engine Based on Flexible Polyimide Memristor

**DOI:** 10.1002/smsc.202200082

**Published:** 2023-01-26

**Authors:** Rui Wang, Saisai Wang, Yuhan Xin, Yaxiong Cao, Yu Liang, Yaqian Peng, Jie Feng, Yang Li, Ling Lv, Xiaohua Ma, Hong Wang, Yue Hao

**Affiliations:** ^1^ Key Laboratory of Wide Band Gap Semiconductor Technology School of Microelectronics Xidian University Xi'an 710071 China; ^2^ Key Laboratory of Wide Band Gap Semiconductor Technology School of Advanced Materials and Nanotechnology Xidian University Xi'an 710071 China

**Keywords:** compressed sensing, compression and encryption, flexible electronics, volatile memristors

## Abstract

It is anticipated that the rapid development of the Internet of Things (IoT) will improve the quality of human life. Nonetheless, large amounts of data need to be replicated, stored, processed, and shared, posing formidable challenges to communication bandwidth and information security. Herein, it is reported that polyimide (PI) threshold‐switching memristors exhibit Gaussian conductance and randomly set voltage distribution with nonideal properties to create a compression and encryption engine with a single chip. The Gaussian conductance distribution is used to achieve compressed sensing (CS) to integrate encryption into compression, and the spontaneous formation of the one‐time‐sampling measurement matrix satisfies absolute security. Moreover, the bitstreams generated by randomly distributed set voltages are used to diffuse the ciphertext from CS to improve security. The engine is shown to be secure even if the eavesdropper knows both the plaintext and the corresponding ciphertext. It has compression performance advantages that take both efficiency and security into account. In addition, due to the superior high temperature and mechanical properties of PI, the engine can continue to function normally in harsh environments. Herein, an excellent solution is offered for ensuring the efficiency and security of IoT.

## Graphical abstract

All‐in‐one compression and encryption engines with one‐time‐only keys constructed from flexible polyimide memristors are achieved, enabling the integration of compression and encryption. The one‐time‐only keys are formed spontaneously to implement compression and diffusion without programming operations, enabling the engine to satisfy perfect security and resist chosen‐plaintext attacks (CPA). An excellent solution is provided for high efficiency and security application.

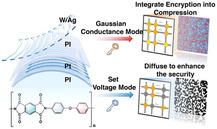

## 1 Introduction

With the advent of the Internet of Things (IoT), numerous perception networks have been constructed to facilitate human–computer interaction. Data must be replicated, stored, processed, and shared in vast quantities. Bandwidth and storage space are becoming increasingly scarce resources. On a daily basis, connected sensing devices are increasingly relied upon to manage vast quantities of digitally classified information and perform security‐sensitive tasks. Nonetheless, hackers can take control of real‐world objects by exploiting security flaws in IoT products. Consequently, compression and encryption of sensory data have become increasingly important. In addition, because each sensor has limited resources and the number of sensors is enormous, a separate cryptographic layer for secure data transmission is too expensive.^[^ ^1^ ^]^ Incorporating a data protection mechanism into the stage of information awareness to achieve simultaneous compression and encryption is a desirable solution.

In the past decade, compressed sensing (CS) has garnered widespread interest in signal processing and wireless communication networks.^[^ ^2^ ^]^ CS uses the structure of specific signals to perform data compression and acquisition simultaneously at the physical interface of the analog and digital domains via a random encoding process, allowing acquisition at sub‐Nyquist rates.^[^ ^3^, ^4^, ^5^ ^]^ Specifically, a Φ‐matrix, also known as a measurement matrix, is used to complete the random encoding process. Any receiver attempting to decode CS measurements must be aware of the actual encoding matrix used during the acquisition to recover the signal accurately.^[^ ^6^ ^]^ Consequently, if the Φ‐matrix is a secret known only to the sender and receiver, it can be used as a key to compress and encrypt the signals simultaneously, and unauthorized users cannot obtain correct sparseness solutions by solving convex optimization problems without knowing the Φ‐matrix, which is a nearly zero‐cost encryption mechanism.^[^ ^7^ ^]^ In recent years, there has been a growing emphasis on CS security. Adopting a one‐time‐sampling (OTS) technique, in which the measurement matrix is never reused, can make the system secure and resistant to chosen‐plaintext attacks (CPA).^[^ ^8^ ^]^ Furthermore, an additional diffusion operation performed by cryptographic primitives can resolve the uneven distribution of CS measurements and withstand statistical attacks. In addition, the hardware implementation of CS for combining compression and encryption has been investigated. In principle, the generation of randomness timing clocks and large‐scale matrix–vector multiplication (MVM) in CS systems consists of complementary metal–oxide–semiconductor circuits and modules.^[^ ^9^ ^]^ While previous research has focused on developing emerging high‐performance sensors, complex sampling control modules, such as OTS generation and high‐intensity MVM operations, continue to be cumbersome, limiting scalability and sampling speed.^[^ ^10^, ^11^, ^12^, ^13^ ^]^ Consequently, a hardware implementation that integrates compression and encryption still faces significant obstacles.

Memristor is one such technology that aims to push future computing beyond Moore's law by developing next‐generation high‐density nonvolatile memory and energy‐efficient in‐memory computing.^[^ ^14^, ^15^, ^16^, ^17^, ^18^, ^19^, ^20^, ^21^ ^]^ In recent years, significant progress has been made in studying new materials for high‐performance memristors, such as transition‐metal oxides and 2D materials.^[^ ^22^, ^23^ ^]^ Compared to inorganic materials, organic polymer memristors have great potential in flexible wearable and implantable medical fields due to their advantages of simple preparation methods, excellent mechanical properties, and biocompatibility.^[^ ^24^ ^]^ Therefore, the development of polymers has necessitated the search for emerging technologies to ensure information security in flexible and wearable fields. In addition, for practical applications, manufacturing variation device‐to‐device (D2D) and intrinsic stochastic cycle‐to‐cycle (C2C) variability, such as resistance variation and probabilistic switching behaviors, could be a major concern.^[^ ^25^, ^26^, ^27^ ^]^ Interestingly, the nonideal effects, which most existing work on memristor‐based systems seeks to eliminate, are precious for CS and cryptosystem research.^[^ ^28^, ^29^, ^30^, ^31^, ^32^ ^]^ In addition, threshold switching (TS) memristors have the ability to automatically return to a high‐resistance state (HRS) and change their internal resistance due to their stochastic behaviors in the formation and rupture of conductive filaments, making them ideal for spontaneously realizing OTS to implement CS and improve its security. To prevent the inability to resist statistical attacks due to the uneven distribution of CS measurements, the random, unrepeatable, and unpredictable key can be generated by the intrinsic physical‐level stochasticity of TS to diffuse the measurements. Notably, the implementation of hardware‐based primitives into software‐based encryption algorithms may be more reliable for next‐generation security applications than the use of potentially vulnerable pseudo‐random number generators. Therefore, the TS is an excellent candidate for compression and encryption integration.

In this work, we developed polyimide (PI) TS memristors to construct an all‐in‐one compression and encryption engine to compress and encrypt sensory data simultaneously, utilizing the inherent variability of the devices, as PI has excellent high‐temperature resistance, mechanical properties, and chemical stability, allowing for widespread applications in flexible and wearable electronics. The successful demonstration of the Gaussian conductance and random set voltage distribution of the PI memristors forms the basis of the engine. The OTS matrix generated by the Gaussian distribution mode is embedded in the information awareness stage as an effective confidentiality layer to integrate encryption into compression, enabling the measurements to meet perfect security requirements and resist CPA. Moreover, the bitstreams generated by randomly distributed set voltages in the array are used as the key to diffuse the measurements to increase security. The current research demonstrates that the hardware all‐in‐one engine can be used to compress, encrypt, and decrypt image data. Even if an attacker is able to freely obtain several plaintexts and their corresponding ciphertexts, the engine itself remains secret.

## 2 Results and Discussion

### 2.1 All‐in‐One Compression and Encryption Engine Based on Flexible Polyimide Memristor

**Figure 1:**
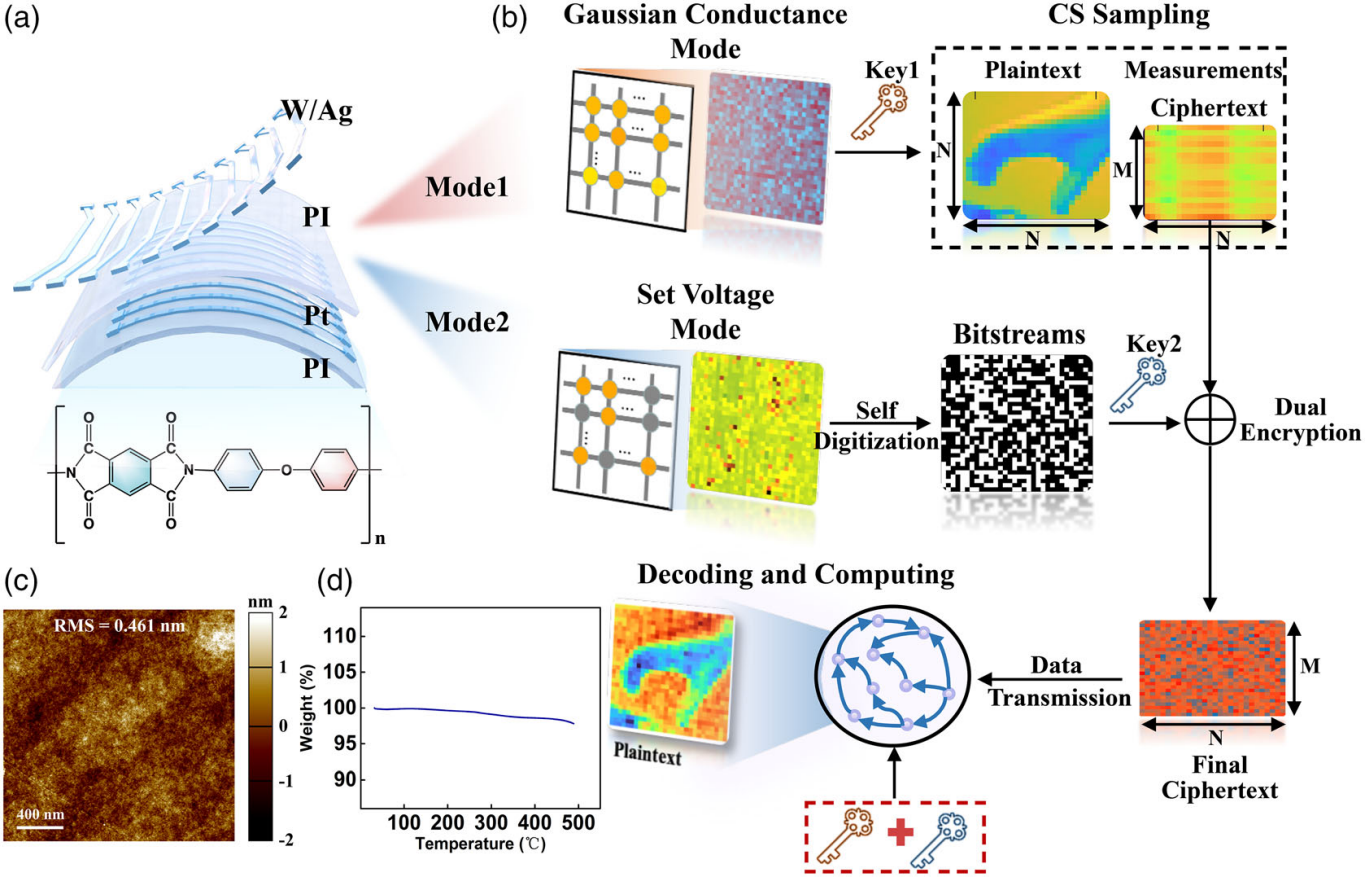
Flexible volatile polyimide memristors for a secure and all‐in‐one compression and encryption engine. a) The structure of polyimide (PI) memristor memristors. b) Schematic representation of the all‐in‐one compression and encryption engine based on PI memristors. Device‐to‐device (D2D) variation in high‐resistance state (HRS) satisfies Gaussian distribution, which can be interpreted as the key for compressed sensing (CS) to simultaneously compress and encrypt. After CS samples the plaintext, the dimension is reduced from *N* × *N* to *M* × *N* (*M* < *N*), and the ciphertext is created. Self‐digitization utilizing leveraging D2D variation in set voltages in large memristor populations to generate a high‐entropy, non‐renewable, and unpredictable key. The key is utilized to diffuse the CS measurements into the final ciphertext. The final ciphertext is eventually decoded and calculated at the receiving terminal, and the keys are exchanged over a secure channel. c) AFM image of the surface of the PI film. The root‐mean‐square (RMS) value of roughness is 0.461 nm. d) PI film thermogravimetric analysis curves.

Traditional CS integrates compression and encryption, but there are security issues such as statistical information leakage, inability to resist CPA, and plaintext outline information leakage. To address the aforementioned issues, the PI memristor was designed to realize a cryptosystem for an integrated compression and encryption engine based on intrinsic oscillatory properties. **Figure** **1**a depicts a schematic representation of the crossbar‐structured tungsten (W)/silver (Ag)/PI/platinum (Pt)/titanium (Ti) on a flexible PI substrate, as well as the molecular formula of PI. Because a large number of fluorine‐containing five‐membered heterocycles and aromatic rings in PI provide strong intermolecular forces and the conjugation effect of aromatic heterocycles provides high thermal stability and mechanical properties, PI enables excellent thermal stability (>500 °C), mechanical toughness, and chemical resistance, thereby providing a stable source of randomness in harsh environments and numerous applications in the flexible and wearable fields.^[^ ^33^, ^34^, ^35^ ^]^ This work specifically exploits two nonideal effects of PI memristors to implement the cryptosystem: Gaussian conductance mode and set voltage mode. For Gaussian conductance mode, all array devices are initially initialized to a low‐resistance state (LRS). The device then returns spontaneously to HRS, and its conductance displays Gaussian random distribution. It is important to note that each time the device returns spontaneously to HRS, the conductance changes compared to the previous one. This means that the measurement matrix is only used once, allowing the cryptosystem to achieve perfect information theory security.^[^ ^8^ ^]^ As demonstrated in Figure 1b, the conductance matrix (measurement matrix) of Gaussian conductance mode is used as a key to suppressing the plaintext from the dimension *N* × *N* to *M* × *N*, and the measurements after CS sampling constitute the ciphertext. Even if the ciphertext provides perfect security, plaintext information can still be revealed. Consequently, the second irrational effect, set voltage mode, is exploited to generate hardware security keys to resolve the issue (Figure 1b). When a voltage with randomly distributed set voltages is applied to the array, the devices can be turned on and set to “1” or left off and set to “0.” Finally, a random bitstream obtained by self‐digitization can be viewed as the key for completing the exclusive‐OR (XOR) operation for diffusion, and the all‐in‐one compression and encryption engine is realized. Notably, the all‐in‐one compression and encryption engine reduces the plaintext size, satisfies perfect security, and conceals the plaintext information exceptionally well, thereby enabling a secure and efficient data transmission. Eventually, the ciphertext is decoded and calculated at the receiving terminal using the secret keys shared by the secure channel. Using atomic force microscopy (AFM), the surface morphology of PI films was characterized, revealing high surface flatness over an area of 2 × 2 μm^2^ with a root‐mean‐square (RMS) roughness value of 0.461 nm (Figure 1c). Thermogravimetric (TG) analysis reveals that aromatic PI has good thermal stability and can remain stable when the temperature reaches 500 °C (Figure 1d), which aids in the development of an all‐in‐one compression and encryption engine that functions well at high temperatures.

### 2.2 Device Characteristics

**Figure 2:**
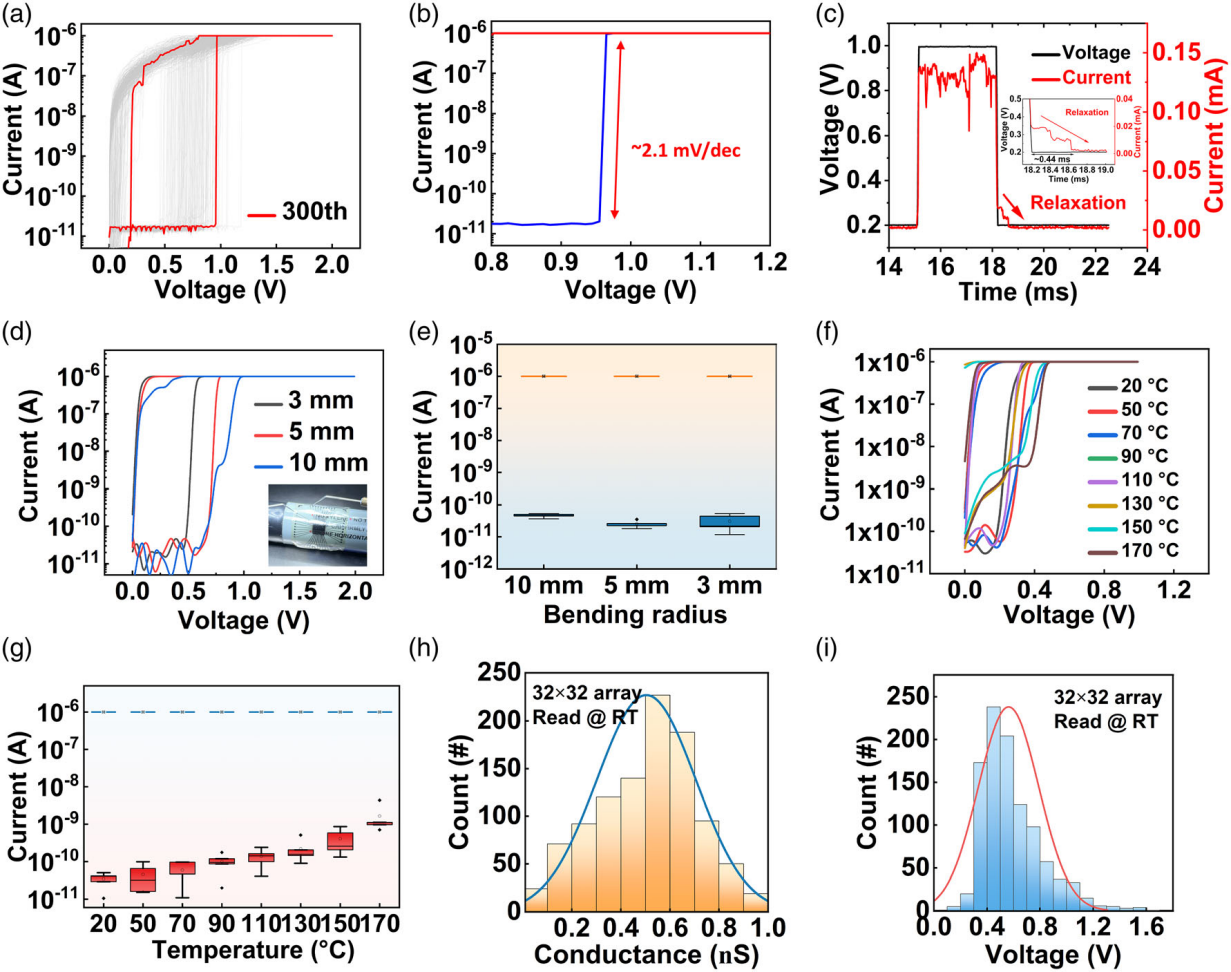
Flexible polyimide memristors. a) *I–V* characteristic curves of the device for up to 300 iterations. b) The device's switching slope is ≈2.1 mV dec^−1^. c) Relaxation time of the device. The relaxation current of the device after a voltage pulse (1 V, 3 ms) is measured using a read voltage of 0.2 V. Inset displays a breakdown of relaxation properties. d) *I–V* curves of the memristor with various curvatures. Inset shows the optical image of the bending test. e) Distributions of on‐state and off‐state currents as a function of curvatures. The standard deviation of five cycles is represented by the error bars. f) Temperature‐dependent *I–V* curves of a memristor. g) Distributions of on‐state and off‐state currents as a function of temperature. The standard deviation of five devices is represented by the error bars. h,i) A statistical histogram of 1024 devices’ conductance and threshold voltages.

The 300 current–voltage (*I–V*) characteristic curves of the device's forming‐free TS are depicted in **Figure** **2**a. Once the applied voltage reaches a threshold, the device switches to LRS, and when the applied potential falls below the holding voltage, the device's resistance rapidly returns to HRS. The *I–V* curve of a PI device with the structure of W/PI/Pt/Ti is shown in Figure S1, Supporting Information. The W/PI/Pt/Ti device showed no resistive‐switching behaviors; therefore, the TS behavior of the W/Ag/PI/Pt/Ti device can also be attributed to the diffusion kinetics of Ag atoms in the resistive‐switching layer. The TS characteristics for different compliance currents (*I*_cc_), ranging from 1–100 μA, are shown in Figure S2a, Supporting Information. The device exhibits a steep turn‐on slope of ≈2.1 mV dec^−1^ (Figure 2b). As depicted in Figure 2c, after a switching voltage pulse of 1 V for 3 ms, the device's relaxation time is approximately 0.44 ms with a read voltage of 0.2 V. Moreover, the transition time to LRS is 300 ns after the device has received the voltage pulse stimulation, as shown in Figure S2b, Supporting Information. Figure S3, Supporting Information, depicts the conductance distribution of HRS under 300 cycles, and it can be seen that the conductance is approximately Gaussian. The behavior of TS devices to spontaneously return to HRS makes it possible to obtain a new conductance matrix for OTS in the array without programming and resetting the conductance of the devices, which is advantageous for conserving energy. To demonstrate the mechanical stability of the concurrent implementation of compression and encryption, the mechanical flexibility of the PI memristor was investigated further. The *I–V* characteristics of the device were measured at 3, 5, and 10 mm bending radii (Figure 2d). Figure 2e depicts the C2C distributions of on‐state (*I*_on_) and off‐state (*I*_off_) currents extracted from five cycles under different bending radii, demonstrating that *I*_on_/*I*_off_ is stable even when the bending radius is 3 mm. As shown in Figure S2c, Supporting Information, when the bending radius of the device is 3 mm, the device can still function normally after being bent 1500 times (Supporting Information). In addition, the device's stability at high temperatures was investigated. Even when exposed to temperatures up to 170 °C, the device maintains a good volatile behavior (Figure 2f). The *I*_on_ and *I*_off_ extracted from 5 devices, and five cycles at different temperatures are shown in Figure 2g and S2d, Supporting Information. At 170 °C, the on/off ratio can reach ≈10^3^. This suggests that PI memristors can perform well even under extreme bending and temperature conditions. The HRS and set voltages of 1024 devices were measured and extracted for histogram statistics to simulate a 32 × 32 memristor array, as shown in Figure 2h,i, respectively, to verify that TS can be used to build the cryptosystem. The statistical histogram of the HRS of 1024 devices demonstrates that when the device returns spontaneously to HRS, the conductance values follow a Gaussian distribution with *μ* = 0.54 nS and *σ* = 0.18 nS. Likewise, these devices display a low and random *V*_TH_ that conforms to a Gaussian distribution with *μ* = 0.56 V and *σ* = 0.23 V. These results successfully validate the nonideal characteristics of PI memristors for the deployment of OTS and random bitstream keys.

### 2.3 Implementation of CS and Security Analysis via Polyimide Memristor

**Figure 3:**
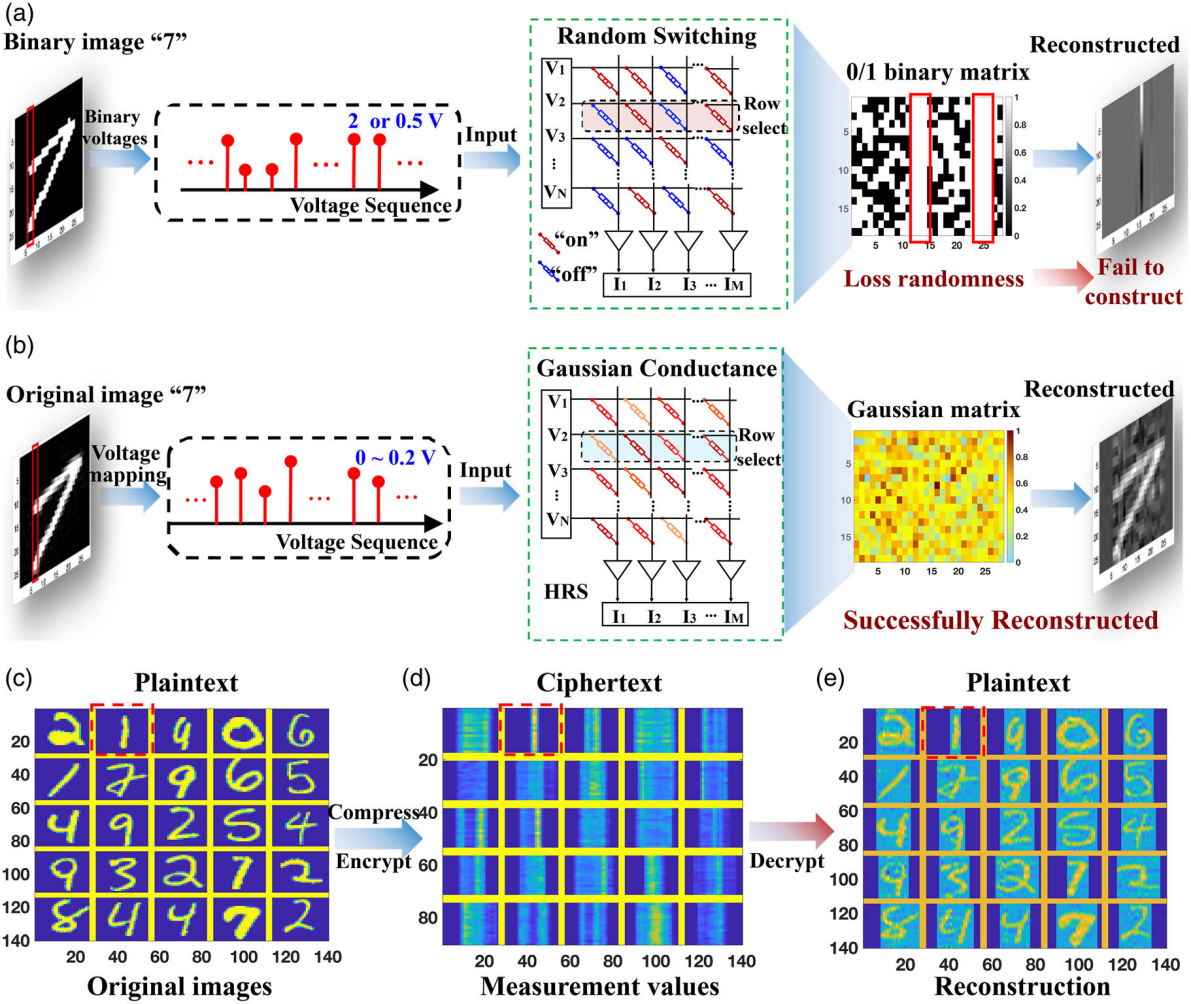
Set voltage and Gaussian conductance mode for CS. a) Set voltage mode for signal compression. The binarized “7” (28 × 28) image is mapped to the voltage sequences of 2 and 0.5 V and applied column by column to the memristor array. Using Kirchhoff's and Ohm's laws, the current is then output in a single step through the column to simultaneously achieve compression and encryption. The orthogonal matching pursuit (OMP) algorithm then reconstructs the signal in the sparse domain. b) Gaussian conductance mode for signal compression. The original image “7” is mapped as a 0–0.2 V voltage sequence and applied to a device array with Gaussian conductance distribution. Signal compression and encryption are achieved using Kirchhoff's current law and Ohm's law. Finally, the OMP algorithm reconstructs the signal. c–e) The original signals (plaintext), the measurements (ciphertext), and the reconstructed signals (plaintext), each with a sampling rate of 60%, respectively.

By assembling devices into a 32 × 32 array using the Gaussian conductance and set voltage mode, it is possible to emulate CS for the Mixed National Institute of Standards and Technology database (MNIST, 28 × 28).^[^ ^36^ ^]^ The crossbar array of the memristor is scalable, allowing it to be used as a measurement matrix to perform MVM operations for data compression.^[^ ^37^ ^]^ Moreover, it is possible to reconstruct the original data from the measurement data. To ensure successful signal recovery, the measurement matrix needs to satisfy the restricted equidistant properties (RIPs) rule. Measurement matrix can be classified into two main categories: random and deterministic. Furthermore, random measurement matrix, such as Bernoulli and Gaussian, is simple to construct and satisfies the RIP with a high probability. In this study, the random measurement matrices generated by set voltage and Gaussian conductance modes were used to realize CS. The matrix needs to satisfy the RIP rule to allow the image to be reconstructed. However, there is no direct algorithm to determine whether the matrix satisfies the RIP property.^[^ ^38^ ^]^ Therefore, the viability of CS based on memristors can only be validated at a practical level (Note S1, Supporting Information). **Figure** **3**a illustrates the set voltage mode for MNIST compression. The image was binarized and converted to voltage sequences (0.5 V as “0”, 2 V as “1”), which were applied to the array to complete the compression process while forming the 0/1 binary measurement matrix. By inputting the voltages of each column into the row of the array, the input signal *X* (*N* × *N*) is multiplied by the measurement matrix Φ (*M* × *N*) to achieve MVM operation, in accordance with Ohm's law and Kirchhoff's equations, and the elements *Y*_*i*_ (*M* × 1) of the measurements *Y* (*M* × *N*) are output in one step through the columns at a sampling rate of *M*/*N* × 100%. The entire procedure can be expressed mathematically as $Y_{i}=\sum_{j}{\text{Φ}}_{ij}X_{j}$. Due to the fact that the MNIST image size is 28 × 28, the Φ is randomly linearly combined with the *X* to reduce the amount of data from 28×28 to 16 × 28 at a sampling rate of 60%. To transform the image to the sparse domain, a discrete cosine transform (DCT) matrix Ψ (28 × 28) satisfying the RIP with Φ was constructed, and the orthogonal matching pursuit (OMP) algorithm was used for image reconstruction.^[^ ^39^ ^]^ Note S2, Supporting Information, describes the process of CS in detail. Sadly, although it is a good idea to perform CS sampling while forming the matrix Φ to save energy and time when programming the matrix, the resulting Φ is dependent on the original signal *X* and lacks randomness, so it is impossible to reconstruct the original signal. Once the randomness is eliminated, there is a high probability that the measurement matrix does not conform to the RIP rule, so the sparse image cannot be restored successfully.

Next, the Gaussian conductance mode for CS is investigated (Figure 3b). First, 2 V electrical pulses are applied to the array to transform the devices into LRS. When the devices return spontaneously to HRS, the conductance exhibits Gaussian distribution, concluding the initialization process. In accordance with the *V*_TH_ of the devices depicted in Figure 2i, the pixel values of the signal are mapped as 0–0.2 V into the array and multiplied by Gaussian conductance in a single step to achieve CS sampling. As stated previously, the HRS must be reconfigured to implement OTS after each column to increase the security of CS sampling. When all columns are subsampled, the signal is compressed and encrypted, which is a nearly cost‐free method of encryption. The power consumption generated by 50% compression of 512 × 512 image is 80 nW. If a one‐time‐only key is considered, necessitating the implementation of OTS, the resulting power consumption is 0.392 mW, which still has a great advantage. As shown in Table S1, Supporting Information, the power consumption of this work is lower than that of the current work. Since the power consumption is mainly generated in the device erasing and writing process, if a nonvolatile memristor is used, an additional voltage operation is required to restore it to HRS, which doubles the power consumption. Specific details of power consumption calculation are presented in Note S3, Supporting Information. The reconstruction method is identical to the set voltage mode (Note S2 and Figure S4, Supporting Information). Since the Gaussian conductance distribution is independent, CS sampling can be performed without destroying the *X*, allowing the *X* to be reliably reconstructed from the measurements Y in sparse domain Ψ. Similarly, the reconstruction result for the digit “7” is depicted in Figure 3b. Although the experimental conductance matrix (Figure 2h) is not a standard Gaussian matrix, the quality of the reconstructed images is unaffected, indicating that the experimentally random conductance matrices satisfy the RIP rule (Figure S5, Supporting Information).

Consequently, the results demonstrate that the Gaussian conductance distribution can effectively implement CS. In addition, the OTS renders the CS‐sampled measurements (ciphertext) compliant with the perfect security of information theory and resistant to CPA (note Figure S4 and S5, Supporting Information). A scenario is examined to elaborate on the secrecy properties of CS measurements. Alice sends Bob a secret signal and selects the key Φ to compress and encrypt signal *X*. Suppose a malicious eavesdropper intercepts ciphertext Y but is unaware of the TS's key Φ from the TS. Is Alice's transmission to Bob secure? Figure S6, Supporting Information, illustrates the pixel histogram statistics of the original image and the “7" ciphertext after CS sampling. The fact that the ciphertext exhibits a clear distribution exposes statistical information to eavesdroppers, who can then launch statistical attacks against it. In addition, due to the implementation of parallel CS, the Φ‐matrix independently compresses each column, which preserves the information of each column of plaintext in the corresponding column of ciphertext, thereby leaking the outline information of plaintext to eavesdroppers. Figure 3c–e depicts, with a sampling rate of 60%, 25 original signals (plaintext), measurements (ciphertext), and reconstructed signals (plaintext), respectively. The image “1,” highlighted by the red line, stands out in particular. The ciphertext of simple signals, such as the number “1,” is not confidential if intercepted by eavesdroppers. The OTS matrix formed by the Gaussian conductance mode can be embedded in the information perception stage as the protection layer to achieve perfect security in a cryptosystem. However, there is a vulnerability to plaintext information leakage. Therefore, the TS's irrational properties are continuously investigated to eliminate these vulnerabilities.

### 2.4 Generation of the Security Key using a Polyimide Memristor

**Figure 4:**
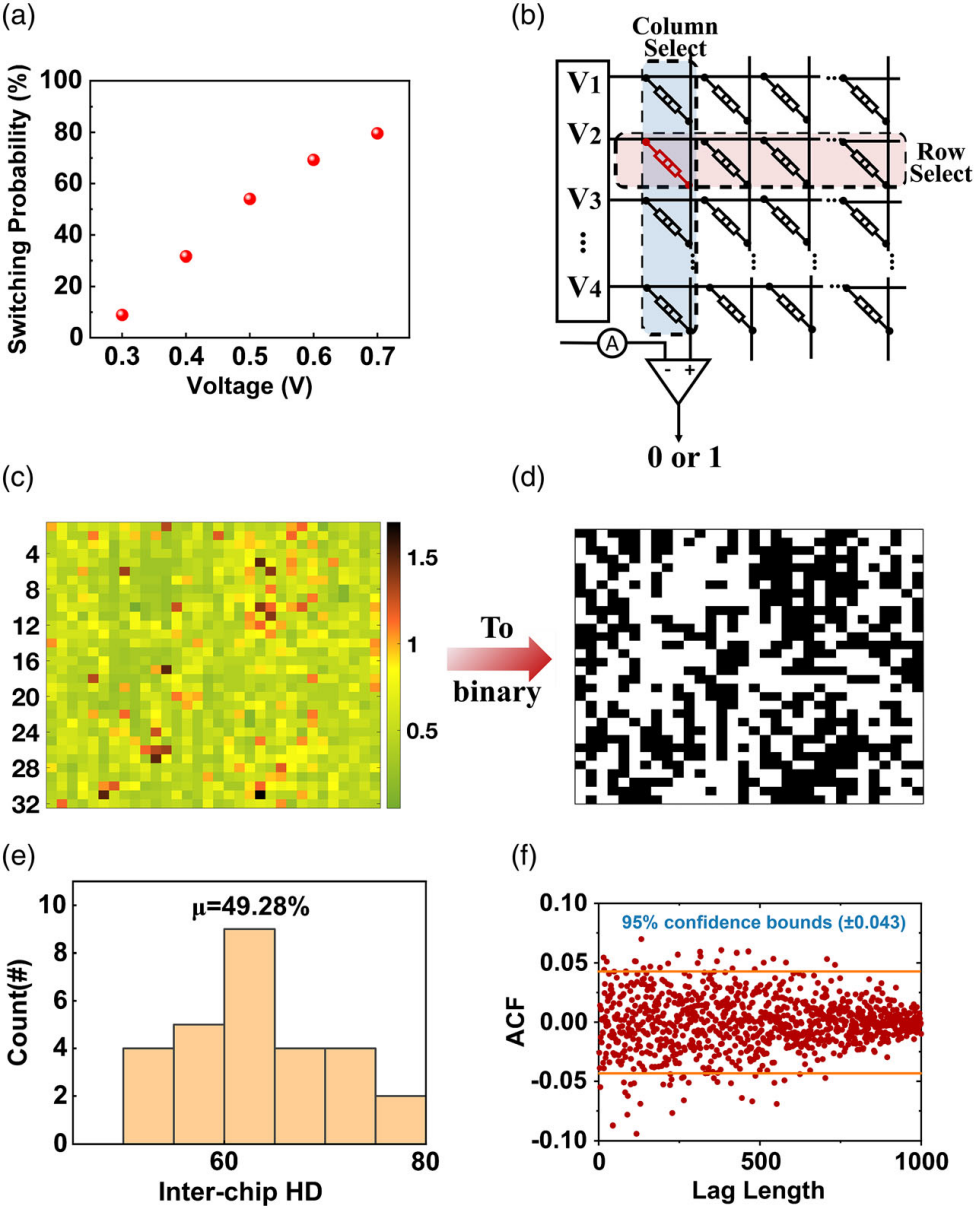
Set voltage mode of polyimide memristor for secret keys generation. a) Probability of device switching as a function of the supplied voltages. b) Circuit architecture consisting of 32 × 32 crossbars for the generation of security keys, with the output of a 0/1 binary matrix passing through a low‐offset comparator. c) An analog map of the *V*_TH_ of a polyimide memristor displaying a high degree of randomness. d) The digital maps acquired after the comparator in (b). e) Inter‐chip Hamming distance (HD) of 8 groups of 128‐bit digital keys. f) Autocorrelation test results for security keys with a 95% confidence level (within ±0.043).

To address the plaintext information leakage vulnerability, a set voltage and Gaussian conductance mode were used to generate random and independent bitstreams for the diffusion operation's security key. For set voltage mode, the device's *I*_off_ is close to 10^−8^ A at 170 °C (Figure 2g), so a threshold current of 10^−7^ A was selected to determine whether each bit is “0” or “1.” **Figure** **4**a exploits the switching probability corresponding to different applied voltages. To increase randomness and achieve a balanced number of “1's” and “0's” in the memristor array, 0.5 V was chosen as the mode's application to the array. The circuit schematic of a simple one‐bit generator is depicted in Figure 4b. The self‐digitization process produces a one‐bit security key output. Notably, the self‐digitization process outperforms the widely employed splitting method, in which reference resistors must be selected and calibrated based on a large number of test devices.^[^ ^40^, ^41^, ^42^ ^]^ Figure 4c illustrates an analog voltage map with a high degree of randomness. As shown in Figure 4d, a 1 kb key is generated in accordance with the binarization described earlier. The inter Hamming distance (HD) has been calculated to validate the randomness and uniqueness of the key.^[^ ^43^, ^44^ ^]^ To calculate the inter‐HD, the array was partitioned into eight groups of 128 devices each. Each group's 128‐bit key was generated using 128 analog device values, and the HD for $\left(\begin{array}{c} 8\\ 2 \end{array}\right)$ = 28 device pairs was calculated. As depicted in Figure 4e, the distribution of Hamming weights is 49.28% and close to 50% with wide variation, indicating the high randomness, independence, and uniqueness of the bit distributions within those keys. Additionally, the HD of the 512‐bit key generated by the Gaussian conductance mode of PI memristors is 47.88%, which is lower than the HD of the key generated by the set voltage mode (Figure S7a,b, Supporting Information). Note S6, Supporting Information, provides additional information and calculation detail on Hamming weight (Supporting Information). Autocorrelation tests further validate the randomness of the keys (Figure 4f), demonstrating that the set voltages of the TS devices are independent variables. Based on these findings, the security keys derived from the set voltage mode of TS devices are highly random and unique for use in future cryptographic applications.

### 2.5 Enhancements to the Security of CS through the Use of Polyimide Memristor

To prevent the measurements from leaking the statistical properties and profile information of the plaintext to eavesdroppers, a method of diffusing measurements involving a bit‐level XOR operation between the quantified measurements and the security keys formed by PI memristors was adopted. Detailed instructions are provided in Note S7 and Figure S8, Supporting Information. Figure S9, Supporting Information, demonstrates that the XOR operation improves CS security. The statistical properties of the number “7” are flattened, whereas the contour information of the number “1” is entirely concealed. It is worth noting that the TS‐generated key is unique and cannot be repeated or predicted. Establishing a secure transmission protocol is also necessary to prevent key exposure and further protect information security. The Diffie–Hellman key exchange protocol, among other things, addresses the secure exchange of secret keys.^[^ ^45^ ^]^ Therefore, even if eavesdroppers intercept the ciphertext without knowing the keys, the ciphertext is secure.

**Figure 5:**
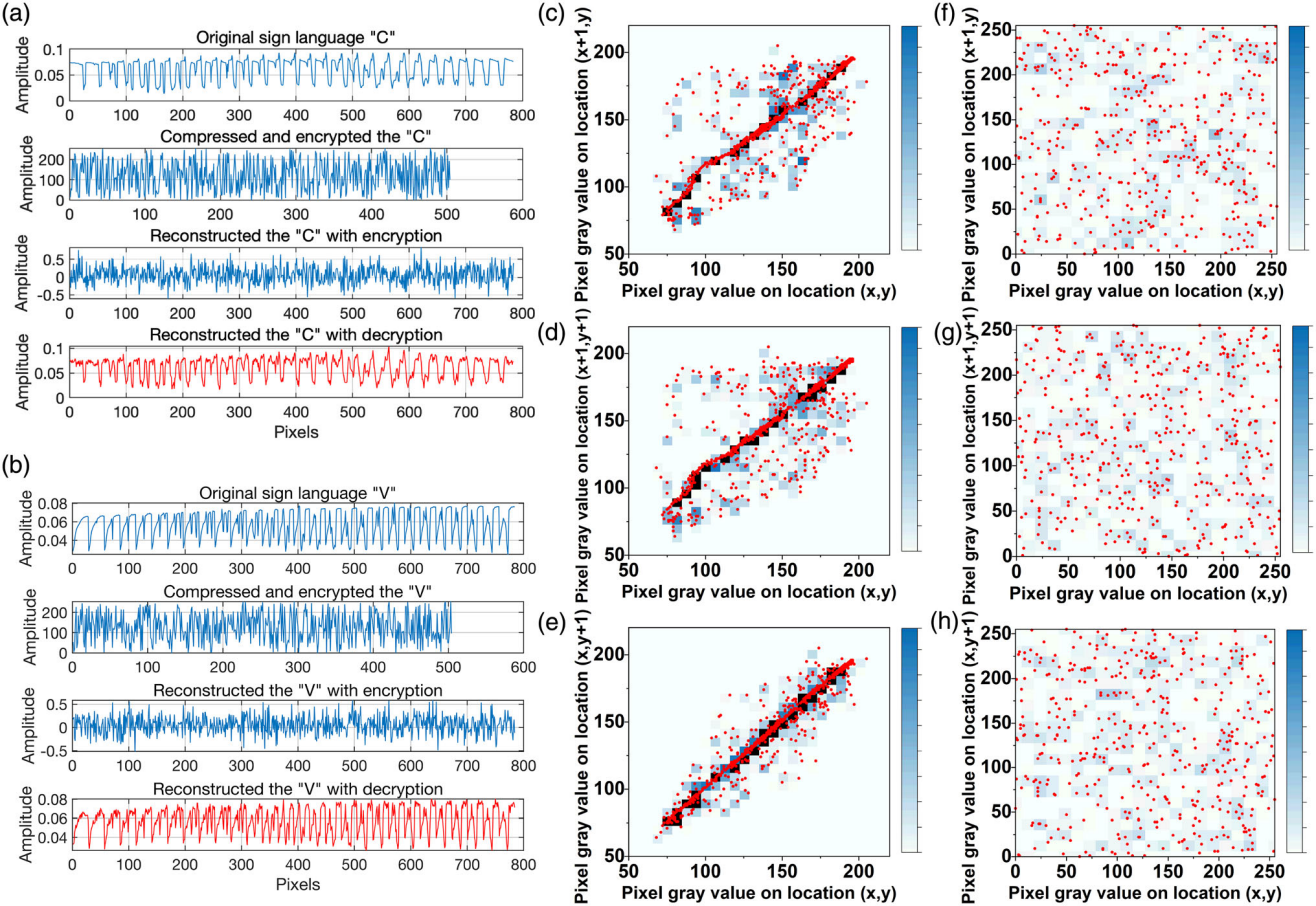
Analysis of the all‐in‐one compression and encryption engine's security. a,b) Original, encrypted compression, encrypted reconstruction, and decryption sign language “C” and “V.” c–h) Correlation distributions of the original image (“V”) and the corresponding encrypted image in horizontal (c) and vertical (f) directions, diagonal (d) and horizontal (g) directions, and diagonal (e) and vertical (h) directions.

The Sign Language MNIST dataset was utilized for verification to demonstrate the scheme's dependability in a practical, complex application.^[^ ^46^ ^]^ The American Sign Language Alphabet Database of Gestures is a multiclass problem with 24‐letter classes (excluding J and Z, which require movement). The dataset contains 27 455 training examples and 7,172 testing examples. Figure S10, Supporting Information, illustrates a selection of sign language data examples. The signals “C” and “V” are encrypted with a 40% reduction in dimensionality, as shown in **Figure** **5**a,b. The cryptosystem has good encryption performance with two keys, and the ciphertext cannot be deciphered without assistance. In addition, since CS is not lossless compression, normalized root mean square error (NRMSE) is used to evaluate the quality of the recovered data.^[^ ^47^ ^]^ NRMSE is defined as follows

(1) $$\text{NRMSE}=\sqrt{\frac{\left\langle ∥\hat{y}\left(t\right)-y\left(t\right){∥}^{2}\right\rangle }{\left\langle ∥y\left(t\right)-<y\left(t\right)>{∥}^{2}\right\rangle }}$$

where $\hat{y}\left(t\right)$ is the recovered signal, *y*(*t*) is the desired signal, ∥⋅∥ denotes the Euclidean norm, and ⟨⋅⟩ denotes the empirical mean. The final successfully decoded signal has a lower NRMSE of 0.23 and 0.25 when the sampling rate is 60%. The outcomes demonstrate that the data were recovered with high precision. In addition, correlation–coefficient analysis was used to assess the efficacy of encryption. Five hundred pairs of adjacent pixels were chosen randomly from the horizontal, vertical, and diagonal directions of the plain and cipher signals. Figure 5c–e depicts the distribution of the original “V” image, while Figure 5f–h illustrates the distribution of the password image. Figure S11, Supporting Information, depicts the corresponding correlation–coefficient analysis of the original image “C” (Supporting Information). The statistical outcomes are displayed in Table S2, Supporting Information. The results demonstrate that the coefficient correlation is substantially reduced compared to the high correlation in ordinary images, and that the information is effectively concealed. Figure S12, Supporting Information, illustrates the statistical histograms of the plaintext, measurements, and final ciphertext of Sign Language “V” (Supporting Information). The distribution of the final ciphertext image is relatively flat, enhancing the secrecy of CS.

**Figure 6:**
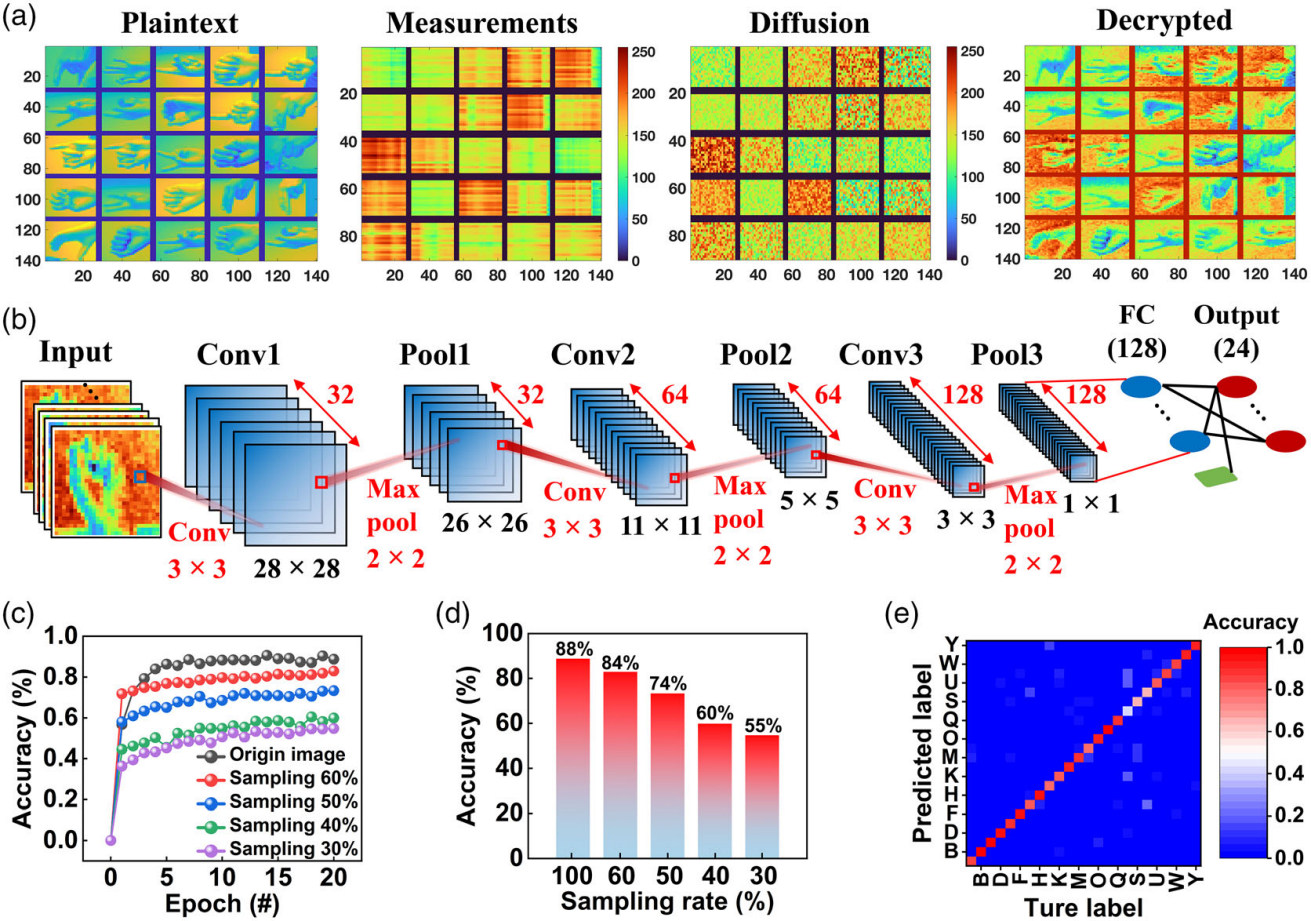
Efficiency evaluation of the all‐in‐one compression and encryption engine. a) From left to right: 25 original plaintexts, the measurement ciphertext of CS sampling, the diffusion results of measurements using the security key of the PI memristors, and the final decryption results, respectively. b) Schematic depiction of the convolutional neural network (CNN) used to identify reconstruction–decryption plaintexts. c) The evolution of recognition accuracy rates throughout 20 epochs with varying sampling rates. d) Rates of recognition accuracy at the 20th epoch for various sampling rates. e) Confusion matrix for classifying the reconstruction–decryption sign language with a sampling rate of 60%.

In addition, the cryptosystem compressed and encoded 25 examples of sign language signals. Plaintexts, measurement ciphertexts, diffused ciphertexts, and decoded plaintexts are depicted in **Figure** **6**a with a sampling rate of 60%. Intuitively, it is clear that the plaintext's information is not only concealed by the diffusion, but also effectively recovered by decoding the ciphertext. The decoded results for sampling rates of 50%, 40%, and 30% are presented in Figure S13a–c, Supporting Information. These results demonstrate the viability of a unified compression and encryption engine. In addition, a convolutional neural network (CNN) with transfer learning was employed to identify reconstruction–decryption plaintexts at various sampling rates (Figure 6b and Note S8, Supporting Information). Figure 6c depicts the training procedure with different sampling rates. Figure 6d depicts the recognition accuracy rates after 20 epochs of training. When the images are subsampled with sampling rates of 60%, 50%, 40%, and 30%, the recognition accuracy reaches ≈84%, ≈74%, ≈60%, and ≈55%, respectively. Figure 6e depicts the confusion matrix for classifying decrypted plaintexts with a sampling rate of 60%, while Figure S13d–f, Supporting Information, depicts the confusion matrices for the remaining sampling rates (Supporting Information). Notably, at a sampling rate of 60%, the recognition accuracy is only 4% lower than the original image, indicating that the cryptosystem can not only compress and encrypt simultaneously, but also reconstruct the plaintext with extremely high recognition accuracy. Consequently, our proposed all‐in‐one compression and encryption engine ensures secure and efficient information exchange.

## 3 Conclusion

We have created a flexible PI TS memristor to construct an all‐in‐one compression and encryption engine for security and efficiency applications. Thanks to D2D and C2C variability, TS devices display Gaussian conductance and fixed voltage mode. The OTS matrix, which is formed spontaneously by Gaussian conductance mode without the need for programming, provides an effective confidentiality layer in the information awareness stage, enabling simultaneous compression and encryption. Since the OTS matrix is unique, the ciphertexts are impenetrable and resistant to CPA. In addition, the security key generated by the set voltage mode is used to diffuse measurements to conceal statistical features and contour data. In addition, the sign language signals were utilized to validate the engine. The results reveal that at a sampling rate of 60%, the recognition accuracy of reconstruction–decryption signals reaches ≈84%, which is only ≈4% less than the accuracy of the original signals; thus, both efficiency and security are considered simultaneously. Due to the PI's superior high‐temperature and mechanical properties, the engine can continue to operate in harsh environments and be utilized in flexible and wearable fields. This work provides an excellent solution for applications requiring high efficiency and security.

## 4 Experimental Section

The flexible TS devices fabricated on prefabricated PI films were as follows: initially, 1000 μL of PI solution and 5000 μL of dimethylformamide (DMF) solution were magnetically stirred at 700 rpm for approximately 12 h at room temperature (RT) to produce a PI‐diluted solution. Then, 10 nm Ti/30 nm Pt was sputtered onto the PI substrate that had been previously prepared as the bottom electrodes. To prepare a dielectric layer, 80 μL of PI diluent was spin‐coated at 4000 rpm onto Ti/Pt/PI, followed by pre‐annealing at 100 °C for 3 min and post‐annealing at 200 °C for 2 h. In the end, 50 nm Ag/50 nm W top electrodes were deposited using Ag and W targets under 100 W direct current (DC) and Ar plasma, respectively. AFM was utilized to analyze the surface roughness of the PI film. Using a TG analyzer, the thermal stability of PI films was analyzed. Keithley 4200 semiconductor parameter analyzer and Agilent 2910A source meter were utilized to measure the electrical behaviors of TS devices.

## Conflict of Interest

The authors declare no conflict of interest.

## Supporting information

Supplementary Material

## Data Availability

The data that support the findings of this study are available from the corresponding author upon reasonable request.
