# Supplementary material for: All‐in‐One Compression and Encryption Engine Based on Flexible Polyimide Memristor
Source: Small Sci. 2023 Jan 26;3(3):2200082. doi: 10.1002/smsc.202200082 (PMC11935910; doi:10.1002/smsc.202200082)
Supplement: Supplementary file 1 — Supplementary Material [file SMSC-3-2200082-s001.pdf]

Supporting Information

All-in-one compression and encryption engine based on flexible polyimide memristor

Rui Wang, Saisai Wang, Yuhan Xin, Yaxiong Cao, Yu Liang, Yaqian Peng, Jie Feng, Yang Li, Ling Lv, Xiaohua Ma, Hong Wang* and Yue Hao*

E-mail: hongwang@xidian.edu.cn
E-mail: llv@xidian.edu.cn

Keywords: volatile memristor, compressed sensing, compression and encryption, flexible electronics

Note S1: Simulation Process of Measurement Process

There is no direct algorithm for compressed sensing to verify whether the measurement matrix satisfies the restricted equidistant properties (RIP), which is a necessary condition for accurate data reconstruction. The data obtained by building a model are usually idealized and challenging to have practical significance. The best solution is to accept completely raw data through hardware entities. In this work, the 1024 conductance distributions are obtained from the experiment formed 32×32 matrix. We experimentally measured the HRS distribution of 1024 devices under multiple cycles. By testing and collecting the conductance values one by one, the random conductance and threshold voltage distribution of the PI memristor were obtained, as shown in Figure 2h, i. The dimension of the compressed image in this article is 28×28; therefore, this experimental data is sufficient to implement the compression process. The measurement process can be represented by the equation. (1-3).

$$Y = \Phi X \tag{1}$$

$$\Phi = \begin{bmatrix} \emptyset_{11} & \emptyset_{12} & \cdots & \emptyset_{1N} \\ \emptyset_{21} & \emptyset_{22} & \cdots & \emptyset_{2N} \\ \vdots & \vdots & \cdots & \vdots \\ \emptyset_{M1} & \emptyset_{M2} & \cdots & \emptyset_{MN} \end{bmatrix} = \begin{bmatrix} \phi_1 \\ \phi_2 \\ \vdots \\ \phi_M \end{bmatrix} \tag{2}$$

$$Y = \begin{bmatrix} y_1 \\ y_2 \\ \vdots \\ y_M \end{bmatrix} = \begin{bmatrix} \emptyset_{11} & \emptyset_{12} & \cdots & \emptyset_{1N} \\ \emptyset_{21} & \emptyset_{22} & \cdots & \emptyset_{2N} \\ \vdots & \vdots & \cdots & \vdots \\ \emptyset_{M1} & \emptyset_{M2} & \cdots & \emptyset_{MN} \end{bmatrix} \begin{bmatrix} x_1 \\ x_2 \\ \vdots \\ x_N \end{bmatrix} \tag{3}$$

where Φ-matrix (including $\emptyset_{11}$, $\emptyset_{12}$, ..., $\emptyset_{MN}$) was obtained from experimental data. And the reconstructed process can be successfully implement in the Φ-matrix. The whole process was implemented using MATLAB.

Note S2: Parallel Compressed Sensing Algorithm Foundation

When the processing object is a two-dimensional signal such as an image, parallel compressed sensing (CS) performs compression and reconstruction on each column of the image through the measurement matrix. Traditional parallel CS usually uses the same measurement matrix for the reduction of each column due to hardware cost and other issues, which is not conducive to taking advantage of the integration of compression and encryption of CS and is easily attacked by malicious eavesdroppers—benefiting from the cycle-to-cycle (C2C) and device-to-device (D2D) changing properties of volatile memristors, a dynamically evolving measurement matrix can be easily implemented for parallel CS.

Assuming that the original signal X in $R^{N\times N}$ is a real-number with the dimension of N × N, which can be regarded as composed of N one-dimensional vectors x_i in $R^{N\times 1}$. Suppose x_i is used to represent the ith column of the X, and Φ with the dimension of M × N represents the measurement matrix. Then, the sub-sampling process of parallel CS can be expressed as:

$$y_i = \Phi x_i \;\; i = 1, \ldots, N \tag{4}$$

where the y_i is the measurements corresponding to each column of X with the dimension of M × 1, and the entire measurements Y are the concatenation of all column measurements y_i, *i.e.*, Y = [y_1, y_2, …, y_N]. For CS, there is a premise that as long as the signal approximately satisfies the sparsity, it can be called a compressed signal, or as long as the signal satisfies the approximate sparsity in a certain transform domain, the signal can be compressed and reconstructed in the sparse domain. Since actual natural images are rarely absolutely sparse, it is often necessary to transform natural images on a sparse basis. $\boldsymbol{\Psi}$ is the sparse orthogonal matrix based on size N × N. Any signal in R_N can be represented by a base of N × 1 vector $\{\psi_i\}_{i=1}^{N}$. Therefore, any signal X can be expressed as:

$$x_i = \Psi s_i, \; i = 1, \ldots, N \tag{5}$$

where s_i is the N × 1 column vector of the weighting coefficients, and the entire weighting coefficients S are the concatenation of all s_i, *i.e.*, S = [s_1, s_2, …, s_N]. X and S are equivalent signals of the exact representation. X is in the time domain, and S is in the $\boldsymbol{\Psi}$ domain. On this basis, the specific CS process for each column can be expressed as:

$$y_i = \Phi x_i = \Phi\Psi s_i = \Theta s_i \tag{6}$$

where the sensing matrix $\Theta = \Phi\Psi$. In addition, the Θ should satisfy the RIP criteria:

$$(1-\delta_k) \parallel x \parallel_2^2 \leq \parallel \Theta x \parallel_2^2 \leq (1+\delta_k) \parallel x \parallel_2^2 \tag{7}$$

where the equidistance constant $\delta_k \in (0, 1)$, k is the number of coefficients. In theory, we can perform a matrix inverse operation to find x ($x = \Theta^T y$), but M is less than N, and the equation is indeterminate. Moreover, solving the $\ell 1$-norm optimization problem is applied to evaluate the signal.

$$\hat{s} = argmin \parallel s' \parallel_1 \text{ such that } \Theta s' = y \tag{8}$$

$$x = \Psi \hat{s} \quad (9)$$

After restoring the images of each column, stitch them together to get the entire original image, *i.e.*, X = [x_1, x_2, …, x_N]. It can be noticed that the sampling and reconstruction processes of parallel CS are processed in a column-by-column manner, which can not only significantly reduce the size of the array but also greatly reduce the computational complexity.

Note S3: Power Consumption of Image Compression

The power consumption is calculated as follows:

$$P = \sum_{i=1}^{N \times M} \frac{V_i^2}{R_i} \quad (10)$$

$$V_i = 0.2 \times \frac{X_i}{255} \quad (11)$$

where V_i is the voltage value of ith input signal; the R_i is the resistance of ith memristor; and the X_i is the pixels value of ith position.

Note S4: Security Analysis of Compressed Sensing

Shannon's research pioneered this approach by introducing the concept of complete secrecy.[1] An encryption scheme can achieve perfect secrecy if the probability of a message conditioned on a cipher is equal to the prior probability of the message $P(X = x|Y = y) = P(X = x)$. Alternatively, this condition can be stated as $I(X;Y) = 0$. Subsequently, the security analysis of CS measurement value is divided into two cases. One is to use a fixed measurement matrix as the key, and the other is to use a dynamically changing measurement matrix as the key. For a fixed measurement matrix, ciphertext is not perfectly secure. This work has theoretically given detailed proof.[2] Details are as follows:

$Proof$: Since the key Φ is a linear measurement, x = 0 means that y = 0. Therefore, $P_{Y|X}(Y = 0 \mid X = 0) = 1$. However, for CS sampling, y is equal to zeros only if X is in nullspace; by an assumption, $P_X(x) > 0$ for all $x \in \mathrm{R}^N$, and $P_Y(Y = 0) < 1$ can be concluded. Therefore $P_{Y|X}(Y = 0 \mid X = 0) \neq P_Y(Y = 0)$, and X and Y are statistically dependent.

For a one-time-only measurement matrix, however, Ciphertext is perfectly secure. The detail is as follows. After defining $\varepsilon_x = \| X \|_2^2$, this work gives the following important result.[3]

Proposition 1: If $\Phi_{i,j}$ are zero-mean Gaussian variables, then CS measurements satisy $I(X;Y) = I(\varepsilon_x;Y)$. According Prop. 1, the following result can be obtained.

Corollary 1: If X ∈ S_β, where S_β = $\{X|\varepsilon_x = \beta > 0\}$, then the CS measurments by one-time-only measurement matrix are perfectly secure.

$Proof$: $If\ \varepsilon_x$ is a prior known constant, then $P(y|\varepsilon_x) = P(y)$. Hence, from Prop. 1 $\mathrm{P}(y|\varepsilon_x) = P(y)$ is stated.

Therefore, using non-volatile memristors is not conducive to realizing secure CS because of the large power consumption required to program the variable conductance matrix. The volatile memristor can easily realize the one-time-only measurement matrix due to the ability to return a high-resistance state spontaneously so that the CS measurements can be perfectly safe.

Note S5: Resistance Attack Analysis of the Gaussian Conductance Mode

Generally speaking, there are four types of classic attacks, namely, chosen-plaintext attacks (CPA), ciphertext only attacks (COA), known-plaintext attacks (KPA), and chosen ciphertext attacks (CCA). In general, CPA is the most powerful attack method. If a cryptosystem can resist CPA, it can also resist other arracks.[4] In classical cryptography, the security of a cryptographic system can only be based on the safety of the key rather than the secrecy of the encryption algorithm or the decryption algorithm itself. Therefore, it is assumed that the attacker is free to obtain multiple plaintexts and corresponding ciphertexts. To resist CPA, the keys in the cryptosystem must be generated dynamically. Otherwise, an attacker can repeatedly construct a special plaintext vector to perform a CPA, thereby cracking the CS encryption scheme. In this Gaussian conductance mode of polyimide (PI) devices, the measurement matrix is one-time-only because of the intrinsic stochastic C2C variability. It is impossible for an eavesdropper to acquire the correct key with the CPA. Accordingly, the one-time-only conductance matrix can be embedded in the information awareness stage as a confidential layer to integrate compression and encryption for a secure and zero-cost cryptosystem.

Note S6: Hamming Weight and Hamming Distance

The Hamming Weight of a key can describe the bit uniformity, which is given as:

$$Uniformity = \frac{1}{n}\sum_{i=1}^{n} k_i \times 100\% \quad (12)$$

where k_i refer the number of a bit “0” of a n-bit key. The ideal proportion of "0" for the entire random response should be 50%, which indicates the numbers of bit “1” and “0” are equal in the key. In this study, n is 1024 and the number of a bit “0” is 472.

The inter-Hamming Distance (inter-HD) is to assess how independent the responses from different encrypted keys are to each other on the chip. The inter-HD is calculated by Equation:

$$Inter\text{-HD} = \frac{2}{m(m-1)}\sum_{i=1}^{m-1}\sum_{j=i+1}^{m}\frac{HD(R_i, R_i)}{n} \times 100\% \quad (13)$$

where R_i and R_j are the n-bit responses of the key “i” and “j” for a given challenge and m is the number of the key and n is the size of the key. In the current work, the *inter-HD* is 49.28% close to 50%. It represents the excellent uniqueness and randomness between different keys.

Note S7: The Process of the All-in-one Compression and Encryption Engine

In this work, signal compression and encryption via Gaussian distributed conductance of the device arrays. At the same time, through a randomly distributed set voltages, the device arrays generate the key to diffuse the measurements. The detail is as follows:

Step 1: Initialize the device arrays to form a Gaussian conductance matrix Φ with the dimension of M × N.

$$\Phi = \begin{bmatrix} \phi_{11} & \cdots & \phi_{1N} \\ \vdots & \ddots & \vdots \\ \phi_{M1} & \cdots & \phi_{MN} \end{bmatrix} \quad (14)$$

Step 2: Convert each column x_i of the plaintext X to voltages and input it into the array. The MVM operation is completed, and the measurements and ciphertext are obtained.

$$y_i = \Phi x_i \quad (15)$$

Step 3: y_i is mapped into the integers of 8-bit by

$$y_i' = round\left[255 \times \frac{y_i - y_{imin}}{y_{imax} - y_{imin}}\right] \quad (16)$$

where round [·] represents rounding procedure, y_i' marked as the result of transforming.

Step 4: Random bitstream keys D_1 are generated through set voltage mode to diffuse the y_i' by XOR operation.

$$D_2 = D1 \oplus y_i' \tag{17}$$

where D_2 is the result of diffusion operation. The decryption process is the reverse of encryption and uses the theory in Note S2 to recover the plaintext from measurements y_i.

Note S8: Architecture of the Convolutional Neural Network

In this study, the CNNs were implemented with Keras to verify the availability of the all-in-one compression and encryption engine. A total of three convolutional layers and pooling layers were utilized. A 3 × 3 size filter was implemented in all the convolution layers, and Max pooling was practiced for the pooling layer. Eventually, the feature plucked from the signal was transported toward the fully connected layer. The activation function of ReLU was simultaneously in the convolution layer and the fully connected layer. Then, the softmax regression was used to fit the weight of the output layer. The optimizer of "Adam" and categorical cross-entropy function were applied in the network with a learning rate of 0.001 and batch size of 128. To bypass overfitting, the dropout method was practiced.

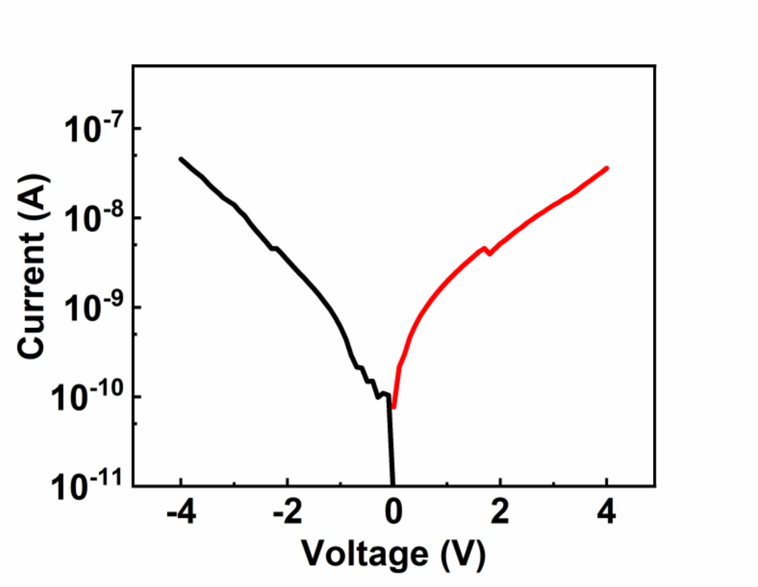

Figure S1. The I-V curve of the PI device with the structure of W/PI/Pt/Ti.

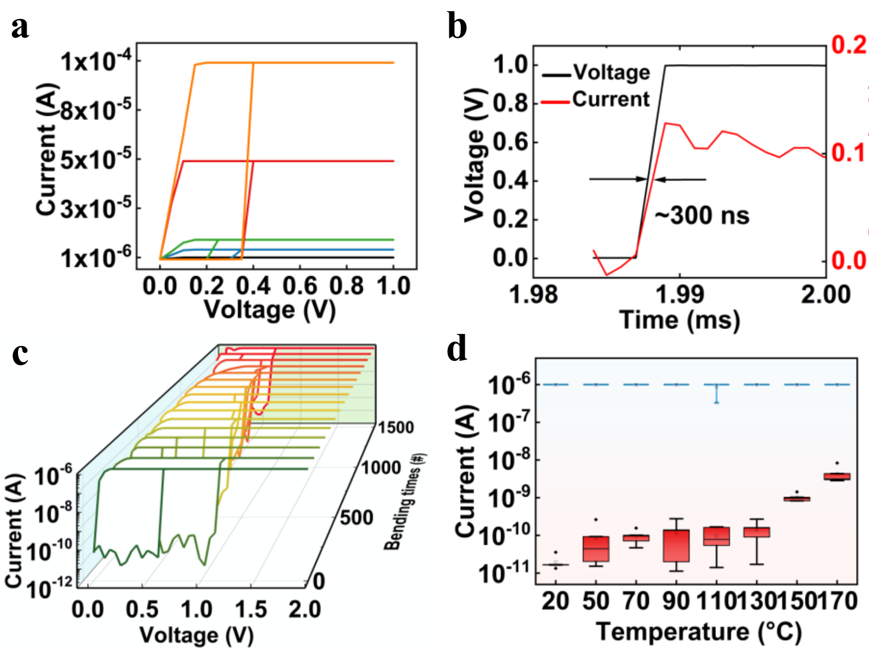

Figure S2. Characteristics of the polyimide memristor. (a) I-V curves of multilevel threshold switching behavior under different I_{cc}. **(b)** Response delay time. **(c)** IV characteristics for 1500 bends with bending radius of 3 mm. **(d)** On-state and off-state current distributions as a function of temperature. Error bars represent the standard deviation of five cycles.

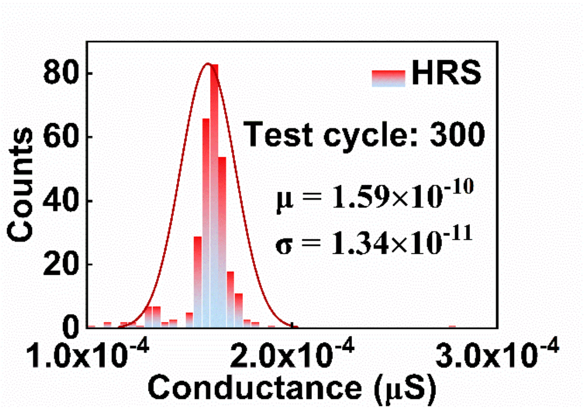

Figure S3. The conductance distribution of HRS with Gaussian fit under 300 cycles.

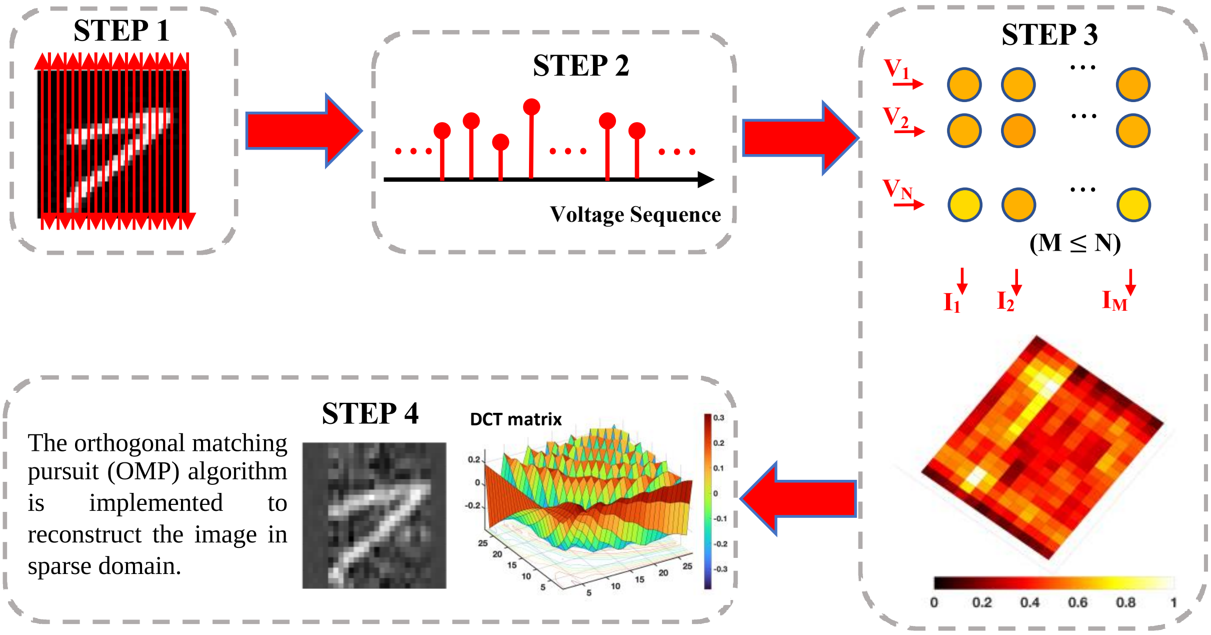

Figure S4. Schematic diagram of CS implementation based on polyimide memristors. The pixel values of the image (N × N) are converted into a sequence of 0 to 0.2 V voltages in columns (STEP 1 and STEP 2). Next, a 2 V electrical pulse is applied to the TS crossbar to make the devices into LRS, and when it spontaneously returns to HRS, the conductance exhibits a Gaussian distribution for measurements matrix Φ. Subsequently, the voltage sequences (N × 1) are applied to the crossbar array (M × N) in columns to achieve matrix-vector multiplication, and the normalized currents (M × 1) are output by Ohm's law and Kirchhoff's law (STEP 3). When all columns are applied to the crossbar array, the compression process is completed, and the compressed signals (M × N) are obtained. Eventually, a discrete cosine transform (DCT) matrix transforms the signals to the sparse domain, and the OMP algorithm is implemented to reconstruct the compressed signals (STEP 4). Notably, illegal users cannot get the correct sparseness solutions by solving convex optimization problems without knowing the Φ-matrix. Therefore, PI crossbar arrays compress and encrypt the signal simultaneously.

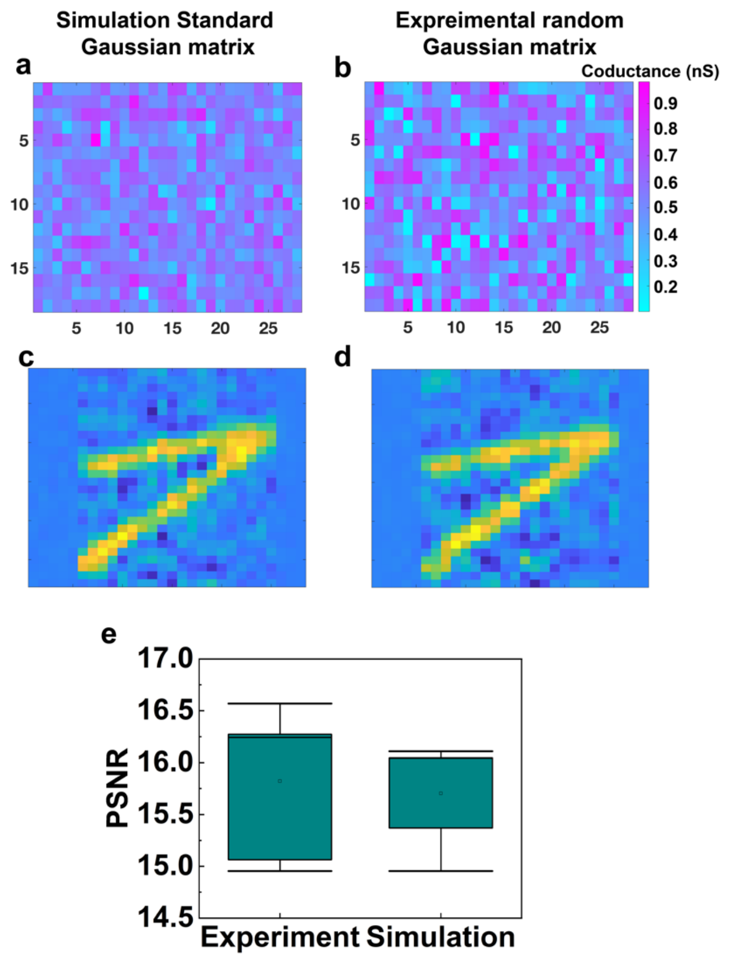

Figure S5. (a-b) The measurement matrix obtained from simulation and experiment respectively. **(c-d)** The reconstructed results from simulation and experiment. **(e)** Recovery performance evaluation of experiment and simulation conductance matrix using PSNR. The error bars stand for standard deviations of 5 CS operations.

Although Figures 2h is not a standard Gaussian distribution, it approximately satisfies the Gaussian distribution and can be fitted with a Gaussian distribution function. The simulation conductance matrix and experimental conductance matrix are shown in Figure S5a, b respectively; the two conductance matrices are used to compress image. The reconstructed results are shown in Figure S5c, d respectively; both matrices can completely restore the image, and the reconstruction results are not significantly different. Furthermore, the standard measurement matrix obtained by simulation and the measurement matrix obtained by experiment to be approximately Gaussian are used to perform 5 CS operations; peak signal-to-noise ratio (PSNR) is used to evaluate the results of CS with simulation and experiment. As shown in Figure S5e experiments and simulations have equal levels of PSNR, which means that the experimentally random conductance matrices satisfy the RIP rule.

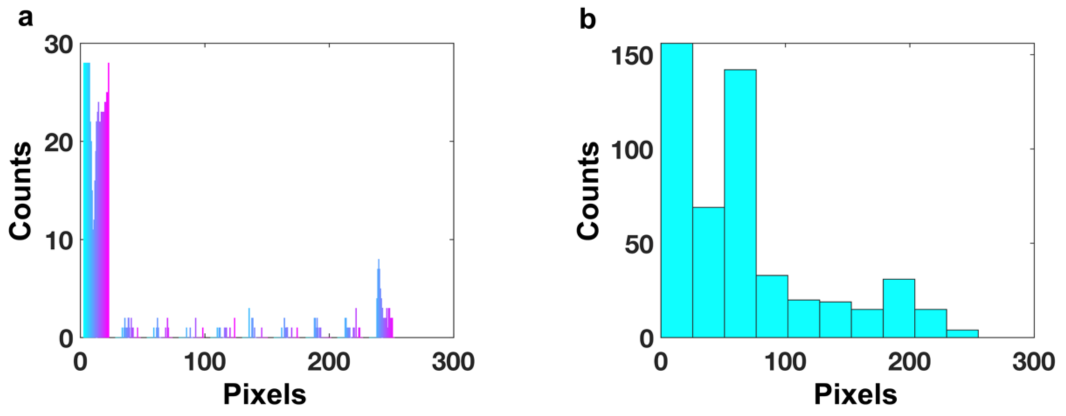

Figure S6. The histogram statistics of the original image **(a)** of "7" and the ciphertext **(b)** after CS sampling.

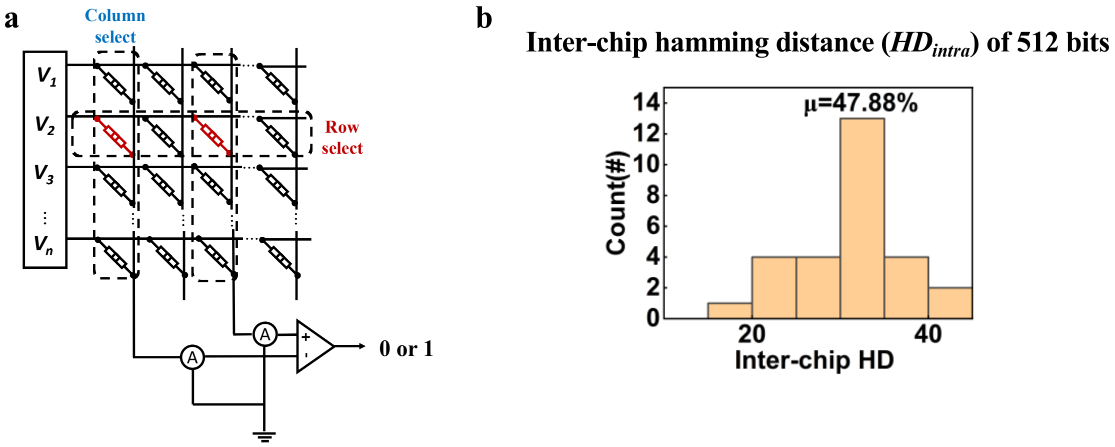

Figure S7. **(a)** Gaussian conductance mode for the generation of a 512-bit key. A one-bit security key output is generated by comparing the total currents running into the two selected paths. **(b)** The inter-chip hamming distance of the 512-bit key generated by the Gaussian conductance mode of PI memristors.

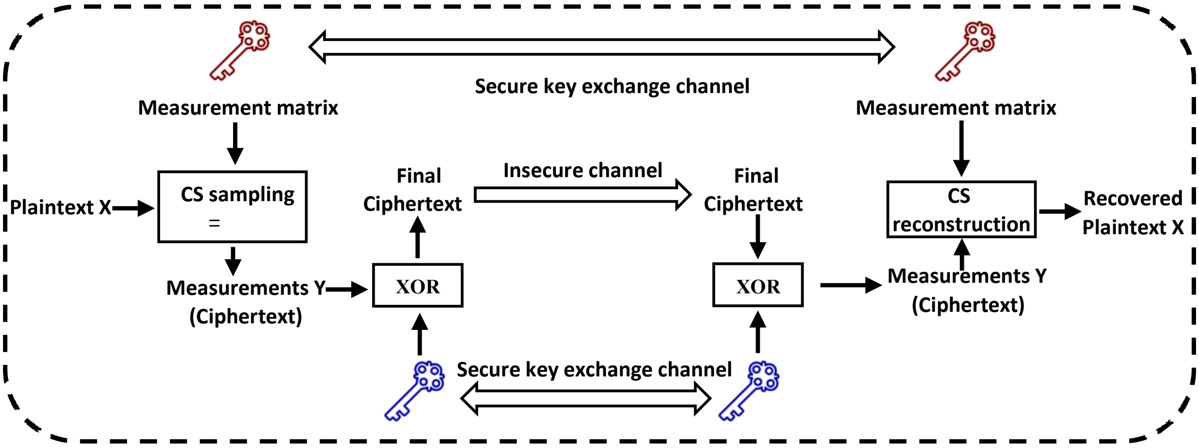

Figure S8. Schematic diagram of the dual encryption CS cryptosystem for the all-in-one crypto and compress engine.

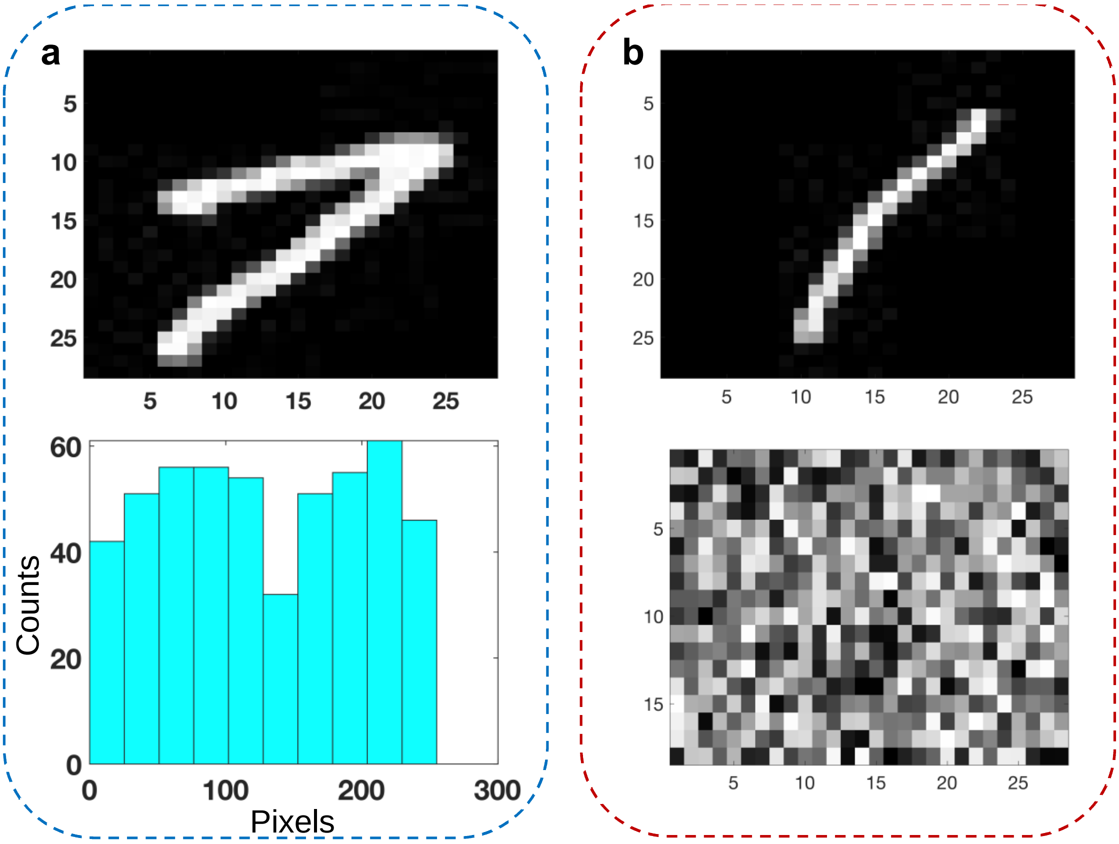

Figure S9. The improvement of the security by the key generated from polyimide memristors. (a) The statistical properties of number "7" after XOR diffusion. **(b)** The measurements ciphertext by CS sampling after XOR diffusion.

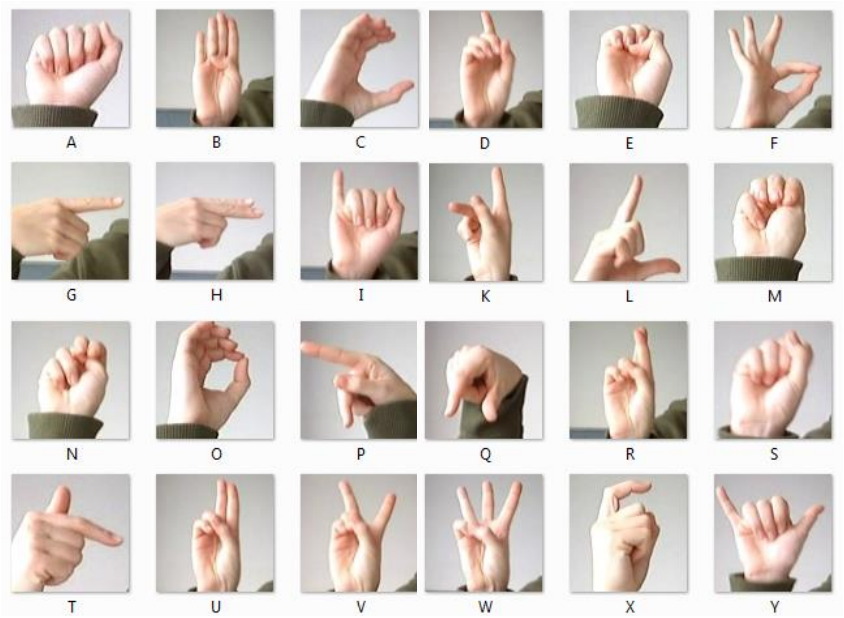

Figure S10. The symbols of the American alphabet at a glance.

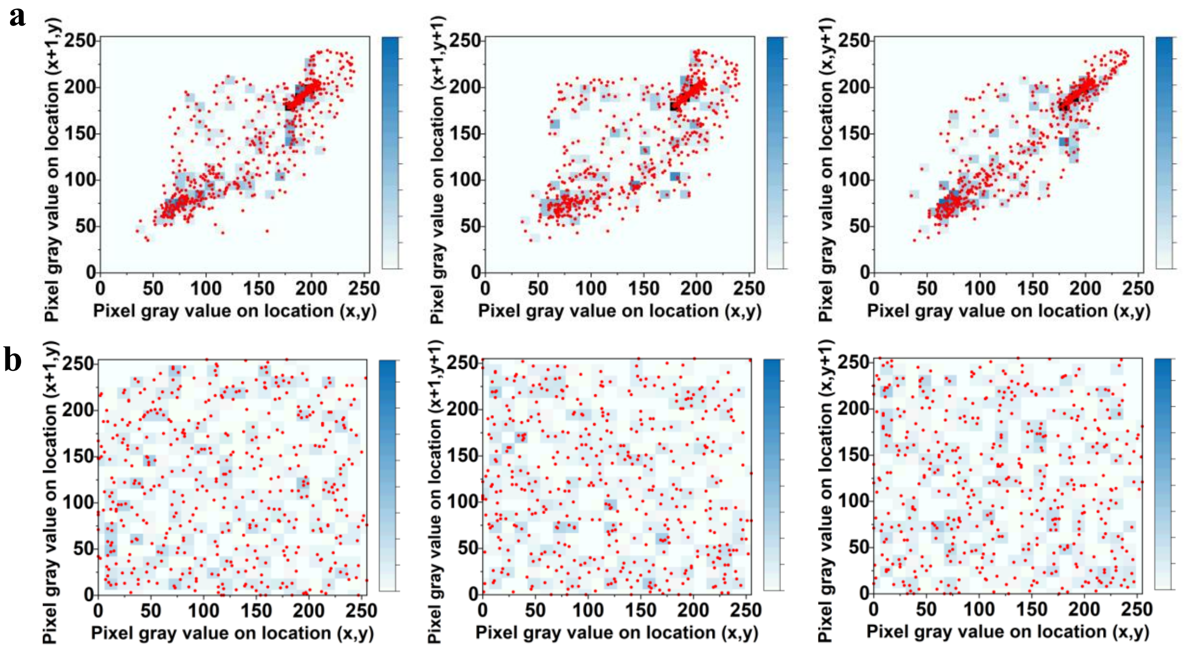

Figure S11. **Pixel correlation analysis of sign language signals. (a)** Correlation distributions of original image "C" in horizontal direction, diagonal direction, and vertical direction respectively. **(b)** Correlation distributions of ciphertext image "C" in horizontal direction, diagonal direction, and vertical direction respectively.

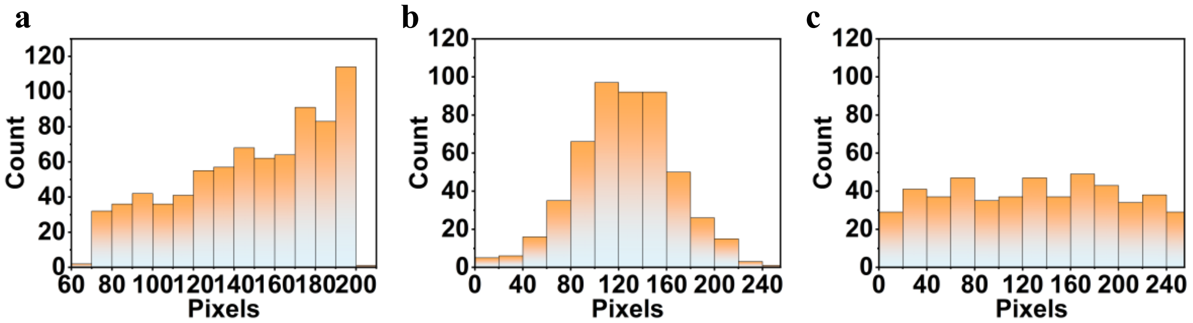

Figure S12. The statistical histograms analysis. The statistical histograms of the **(a)** plaintext, **(b)** measurements and **(c)** final ciphertext of Sign Language "V".

Sampling Rate:

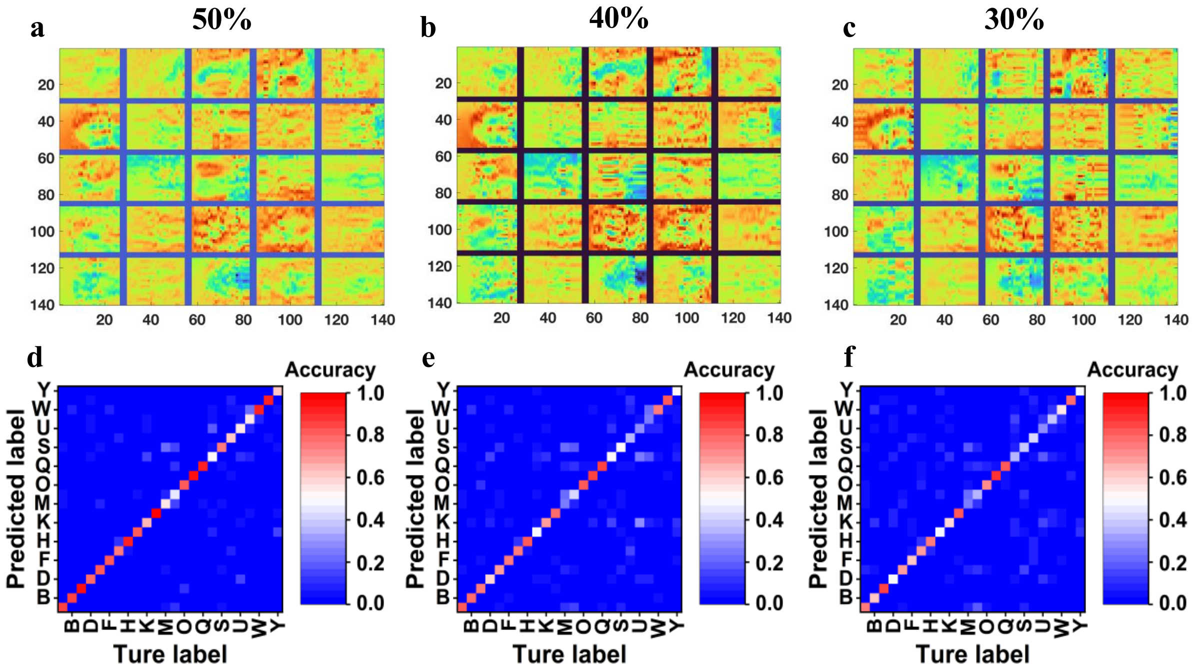

Figure S13. **Efficiency evaluation of sign language signals with different sampling rates.** **(a-c)** The decrypted and reconstructed data with the sampling rate of 50%, 40%, and 30%. **(d-f)** The confusion matrix with the sampling rate of 50%, 40%, and 30%. The recognition accuracy reaches 74%, 60%, and 55% respectively.

Table S1. Comparison between different compress engines.

**Technology**	**Image dimension**	**Power**	**Reference**
FPGA	256×256	800 mW	[5]
PCM	256×256	16.2 mW	[6]
TiOx non-volatile memristor	-	18.45 nW	[7]
PI volatile memristor	512×512	80 nW	This work

Table S2. Correlation coefficients of original and encrypted signals.

Image	Horizontal	Vertical	Diagonal
Original "V"	0.9678	0.8155	0.7561
Encrypted "V"	0.0556	-0.0777	-0.0755
Original "C"	0.9180	0.8842	0.7890
Encrypted "C"	0.0270	0.0181	0.0217

Reference

[1] C. E. Shannon, *Bell Syst. Tech. J.* **1949**, *28*, 656.

[2] Y. Rachlin, D. Baron, presented at *2008 46th Annual Allerton Conf. Communication, Control, and Computing*, IEEE, Monticello, Illinois, USA **2008**.

[3] T. Bianchi, V. Bioglio, E. Magli, *IEEE Trans. Inf. Forensics Secur.* **2016**, *11*, 313.

[4] L. Xue, Y. Wang, Z. Wang, *IET Inform. Secur.* **2022**.

[5] M. Le Gallo, A. Sebastian, G. Cherubini, H. Giefers, E. Eleftheriou, presented at *2017 IEEE Inter. Electron Devices Meeting (IEDM)*, IEEE, San Francisco, California, USA **2017**.

[6] M. Le Gallo, A. Sebastian, G. Cherubini, H. Giefers, E. Eleftheriou, *IEEE Trans. Electron. Dev.* **2018**, 65, 4304.

[7] F. Qian, Y. Gong, G. Huang, K. Ahi, M. Anwar, L. Wang, presented at *2016 IEEE/ACM Inter. Symp. on Nanoscale Architectures (NANOARCH)*, IEEE, Beijing, China **2016**.
